# Improvement of Biomineralization of *Sporosarcina pasteurii* as Biocementing Material for Concrete Repair by Atmospheric and Room Temperature Plasma Mutagenesis and Response Surface Methodology

**DOI:** 10.4014/jmb.2104.04019

**Published:** 2021-08-03

**Authors:** Pei-pei Han, Wen-ji Geng, Meng-nan Li, Shi-ru Jia, Ji-long Yin, Run-ze Xue

**Affiliations:** 1 State Key Laboratory of Food Nutrition and Safety, Key Laboratory of Industrial Fermentation Microbiology, Ministry of Education, College of Biotechnology, Tianjin University of Science and Technology, Tianjin 300457, P.R. China; 2 Tianjin Research Institute for Water Transportation Engineering, M.O.T., Tianjin 300456, P.R. China

**Keywords:** Biomineralization, calcite, calcium carbonate, concrete repair, optimization

## Abstract

Microbially induced calcium carbonate precipitation (MICP) has recently become an intelligent and environmentally friendly method for repairing cracks in concrete. To improve on this ability of microbial materials concrete repair, we applied random mutagenesis and optimization of mineralization conditions to improve the quantity and crystal form of microbially precipitated calcium carbonate. *Sporosarcina pasteurii* ATCC 11859 was used as the starting strain to obtain the mutant with high urease activity by atmospheric and room temperature plasma (ARTP) mutagenesis. Next, we investigated the optimal biomineralization conditions and precipitation crystal form using Plackett-Burman experimental design and response surface methodology (RSM). Biomineralization with 0.73 mol/l calcium chloride, 45 g/l urea, reaction temperature of 45°C, and reaction time of 22 h, significantly increased the amount of precipitated calcium carbonate, which was deposited in the form of calcite crystals. Finally, the repair of concrete using the optimized biomineralization process was evaluated. A comparison of water absorption and adhesion of concrete specimens before and after repairs showed that concrete cracks and surface defects could be efficiently repaired. This study provides a new method to engineer biocementing material for concrete repair.

## Introduction

Due to its abundance, low cost and simplicity of production, concrete as a raw material is one of the most widely used in construction, making it indispensable in modern civil engineering. Nevertheless, due to constant loads and external factors, cracks inevitably appear during the long service life of concrete in different environments, reducing the durability of concrete structures. Traditional repair method are limited due to their high cost and the complexity [1], and therefore, alternative repair technologies are urgently needed [2, 3].

Biomineralization is a common phenomenon in nature. With the participation of living cells, inorganic elements can be selectively precipitated from the environment on specific organic matter to form biominerals. MICP is the most common type of biomineralization [4, 5]. The calcium carbonate formed by microbial mineralization has high strength, excellent fracture toughness, good cementation, and excellent compatibility with cement-based materials. Moreover, the process is highly controllable, and a large amount of calcium carbonate can be produced in a short time. Therefore, MICP technology is used in the repair of concrete structures to improve their durability [6-9]. *Sporosarcina pasteurii* is a commonly used carbonate mineralized bacterium with high urease activity that can induce calcium carbonate precipitation in the presence of urea. The reaction mechanism is as follows [10-12]:

$${\text{CO(NH}}_{2})+{\text{H}}_{2}\text{O}\overset{\text{urease}}{\to }{\text{NH}}_{2}{\text{COOH+NH}}_{3}$$

$${\text{NH}}_{3}{\text{COOH+H}}_{2}\text{O}\to {\text{NH}}_{3}+{\text{H}}_{2}{\text{CO}}_{3}$$

$${\text{H}}_{2}{\text{CO}}_{\text{3}}↔{\text{HCO}}_{3}^{-}{\text{+H}}^{\text{+}}$$

$${\text{2NH}}_{\text{3}}{\text{+2H}}_{\text{2}}\text{O}↔{\text{2NH}}_{\text{4}}^{\text{+}}{\text{+2OH}}^{\text{-}}$$

$${\text{HCO}}_{\text{3}}^{\text{-}}{\text{+H}}^{\text{+}}{\text{+2OH}}^{\text{-}}↔{\text{CO}}_{\text{3}}^{\text{2-}}{\text{+2H}}_{\text{2}}\text{O}$$

$${\text{Ca}}^{\text{2+}}{\text{+CO}}_{\text{3}}^{\text{2-}}↔{\text{CaCO}}_{\text{3}}\text{.}$$

Biomineralization refers to the process of constructing a hierarchical structure based on inorganic minerals under certain environmental conditions. This process is highly regulated by the biological microenvironment. Although microbial mineralization technology has attracted great interest from the fields of science and technology, most of the research results are far away from actual application [13]. There are problems such as low efficiency of microbial mineralization, complex environment, wide changes of pH, and complex pollutants, which all reduce the effectiveness of microbial mineralization in practical applications. Additionally, the mineralogy and morphology of precipitates are also important parameters that need to be considered. Calcium carbonate can be deposited in the form of calcite, vaterite and aragonite, which all have different physical properties, such as solubility, density and hardness, and can significantly influence the final concrete repair properties [5]. Among them, calcite has the greatest stability, the highest density and the most compact structure, making it the most suitable for concrete repair. A few studies have investigated ways to control the morphology of inorganic crystals [14, 15], but there is still a lack of relevant reports on the yield optimization of the desired crystal form in the study of microbial remediation of concrete cracks [16, 17].

It is difficult to use conventional methods to repair the structural damage to concrete in practice. However, microbially induced calcium carbonate precipitation can achieve a good repair effect on concrete cracks, since the precipitated calcium carbonate also has excellent compatibility with cement-based materials because of its good cementing effect. However, the key to the success of this method is whether a sufficient amount of calcium carbonate can be produced. In this study, the test strain was subjected to mutagenesis to increase its urease activity, so as to improve its ability to induce calcium carbonate precipitation. Furthermore, the influence of mineralization conditions on MICP was analyzed, and the mineralization conditions were optimized to further improve the ability of the engineered strain to achieve the desired result. Finally, the concrete repair effect of the optimized process was evaluated.

## Materials and Methods

### Microorganism and Culture Conditions

The starting strain used in this study, *S. pasteurii* ATCC 11859, was purchased from the American Type Culture Collection and grown at 30°C and 200 rpm NH_4_-YE (yeast extract 20 g/l, (NH_4_)_2_SO_4_ 10 g/l, and 0.13 mol/l Tris-HCl (pH 9.0). The primary plate screening medium was composed of yeast extract 20 g/l, (NH_4_)_2_SO_4_ 10 g/l, 0.13 mol/l, Tris-HCl (pH 10.0), and agar powder 20 g/l. The mineralization medium consisted of calcium chloride 0.75 mol/l, and urea 50 g/l at pH 7.0.

### ARTP Mutagenesis Procedure and Screening of Mutant Strains

Cells in the exponential growth phase were collected and washed three times with sterile PBS buffer (pH 7.0, 0.1 mol/l) and resuspended to a concentration of 10^6^~10^8^ cells/ml. Then, the mutagenesis was performed using an Atmospheric and Room Temperature Plasma instrument (ARTP, Siqingyuan, China) at a power setting of 100 W, helium flow of 10 SLM (standard liters per min), operating distance of 2 mm, and plasma temperature of 30°C. For the treatments, 10 μl of the bacterial suspension was uniformly applied to the center of the carrier under sterile conditions and then subjected to the plasma jet for several time intervals including 20 s, 25 s, 30 s, 35 s, 40 s, and 45 s with 0 s as the control.

After the mutagenesis was completed, sample plates were washed with PBS buffer, diluted in an appropriate gradient, and 100 μL of the bacterial solution was spread on the screening plates. After culture, larger colonies with a faster apparent growth rate were picked and seeded into NH_4_-YE liquid medium. The strain with the highest urease activity was selected for further experiments.

The urease activity was determined by the conductivity method as previously reported [16]. The urease enzyme activity was measured by calculating the amount of hydrolyzed urea using the equation:

Urea hydrolysis (mM) = conductivity change (ms/cm) ×11.11 (R^2^ = 0.9988).

The determination of urease activity was performed by measuring the change of conductivity (FE30, Switzerland) within 5min after mixing 1 ml of bacterial solution and 9ml of 1.11M urea solution. The enzyme activity calculation formula [18,19] was as follows:

$$\text{Urease activity (mM/min) = }\frac{\text{Conductivity change value}}{5}\times 11.11\times 10$$

$${\text{Unit enzyme activity (mM/(min/OD}}_{600}))=\frac{\text{Urease activity}}{{\text{OD}}_{600}}.$$

### Single Factor Experiments

In the single factor experiment, the amount of calcium carbonate was used as the indicator to investigate the effects of mineralization conditions including calcium chloride, urea, nickel chloride, ammonium sulfate, yeast extract and temperature on the biomineralization process. The initial conditions for biomineralization in a 50 ml reaction mixture were as follows: 0.75 mol/l calcium chloride, 50 g/l urea, reacting at 35°C for 18 h with pH 7.0, and the cell concentration kept at at 7.0 × 10^6^ cells/ml. When investigating a single factor, the remaining factors are fixed. Each factor was tested at 5 levels in the following ranges: calcium chloride 0.25-1.25 mol/l, urea 20-60 g/l, nickel chloride 0-1.0 μmol/l, ammonium sulfate 0-12 g/l, yeast extract 0-12 g/l, and temperature of 30-50°C.

### Experimental Design and Statistical Analysis

The Plackett-Burman experimental design with N=12 at two levels (-1, 1) was used to screen the factors that have significant effects on the biomineralization process using Minitab17 software (Minitab Inc., USA).

To estimate the optimal levels of significant factors, the steepest climbing test was used to quickly approximate the optimal value region, thereby determining the central point of response surface optimization [1].

Based on the Plackett-Burman test and steepest climbing test, the response surface methodology was used to determine the optimal value for the mineralization conditions. Using the Box-Behnken design principle in Design-Expert 8.0.5b (State-Ease Inc., USA) software, a three-factor and three-level design was carried out, in which three central test points and 12 factorial points were set. A total of 15 experiments were performed with three replications [20].

### Extraction, Characterization, and Quantification of Microbially Precipitated Calcium Carbonate

After the end of the mineralization reaction, the fermentation broth was removed, and the obtained precipitated material was washed with sterile water, dried at 60°C overnight, and stored for use.

To extract the calcium carbonate, the fermentation broth was passed through a vacuum filter with a 0.2-μm pore-size membrane (Advantec, Japan). The precipitates were subsequently washed three times with distilled water and oven dried overnight at 60°C. The final pH and absorbance of each medium was measured prior to filtration using a conventional pH Meter (PHSJ-4A, Lei-ci Instruments, China) and spectrophotometer (Shimadzu, UV-1700, Japan) at 600 nm, respectively.

The surface morphology was observed using a scanning electron microscope (SEM, Hitachi S-4800, Japan), and the scanning microdomains were subjected to elemental analysis using an Energy Dispersive Spectrometer (EDS, X-Max 20, UK) system.

Samples were prepared by KBr solid tableting method and analyzed by Fourier transform infrared spectroscopy (FTIR, Nicolet iS50, USA) with a resolution of 4 cm^-1^ and scanning range of 4,000-400 cm^-1^.

X-ray diffraction (XRD-6100, Shimadzu, Japan) was used analyze the crystal forms of the precipitated calcium carbonate. The scanning range (2*θ*) was adjusted from 10° to 80°. The step size and speed were 0.02°, and 2°/min, respectively.

### Analysis of the Crystal Forms of the Microbially Precipitated Calcium Carbonate

The quantification of calcium carbonate crystal form was performed by XRD using an internal standard method. Pure vaterite and calcite was synthesized according to the method described by Mori [21]. Various ratios of calcite and vaterite were mixed, and the relationship between the two components was expressed using the following formula [22]:

$$\frac{I_{C}}{I_{V}}=\wedge \times \frac{X_{C}}{X_{V}},$$

where *I_C_/I_V_* is the peak integrated area ratio of the diffraction peak of calcite (104) to that of vaterite (110), and *X_C_/X_V_* is the molar fraction ratio of calcite to vaterite.

The constructed standard curve was y=4.130x+0.107 (R^2^=0.9999), where y is the amount of precipitated calcium carbonate, and x is the calcium carbonate of different crystal forms.

### Concrete Repair Test

Cylindrical concrete specimens with surface defects and cracks of different sizes were prepared. Then, the mutant strains were cultured in NH_4_-YE liquid medium, collected by centrifugation, and resuspended in saline solution. The surface defects of the concrete samples were repaired by agar solid bacteria surface brushing. The nutrient solution containing 15 g/l agar, 45 g/l urea, 0.75 mol/l calcium chloride and 0.25 μmol/l nickel chloride was heated and dissolved. After adding the bacterial suspension, a total of 10 ml was evenly brushed onto the surface of the specimens. For the cracks, the repair method of pre-adding quartz sand and then pouring in bacteria liquid and nutrient salt was used. The concentration of bacteria was adjusted to 7.0 × 10^6^ cells/ml with the nutrient salt solution. The cracks were filled with quartz sand, and then the nutrient salt solution containing bacteria cells was added into the cracks until they were fully filled. The samples obtained from the above operation were used as the experimental group. In the control group, specimens with surface defects and cracks were both repaired without adding bacteria while other conditions remained the same as above. Then, the specimens were placed in an incubator (DH-204, Zhonghuan, China) at 45°C. The operations were repeated every 12 h for 7 days continuously.

The repair efficiency of concrete specimens was mainly characterized by quantifying water absorption and adhesion. For the water absorption measurement, the specimen was first dried to a constant weight in an oven at 100°C, the weight was recorded as X_1_, then after fully soaking the repair surface in water to saturate it, the specimen was weighed immediately and recorded as X_2_. The water absorption (%) was calculated as (X_2_ - X_1_)/X_1_ × 100%. The adhesion of the specimens was assessed by measuring the loss of mass after ultrasonication.

## Results 

### Screening of Improved Mutants

*Sporosarcina pasteurii* ATCC 11859 was used as the starting strain for ARTP mutagenesis, and the lethality rate of the strain was calculated under different treatment times (Fig. S1). After 30 s of treatment, the lethality rate was 91.06%, which was close to the 90% fatality rate recommended in previous studies [23, 24]. To make the mutation become positive, and considering the stability of the mutation, accordingly, 30 s was chosen as the appropriate mutagenesis treatment time.

**Fig. 1:**
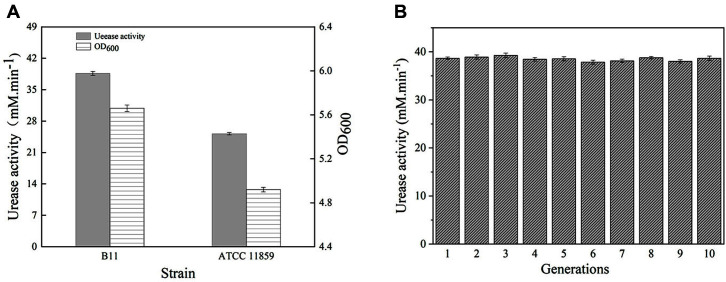
(**A**) Comparison of growth and urease activity between *Sporosarcina pasteurii* ATCC 11859 and mutant B11. (**B**) The genetic stability of mutant B11.

A total of 280 mutant strains with improved traits (Fig. S2) were selected on the primary screening plate for the subsequent shake-flask fermentation screening. After an initial shake-flask screening and re-screening, six strains with higher urease activity were selected (Table S1). According to the urease activity and unit urease activity, the mutant B11 was found to be the most promising (Fig. 1A). The mutant B11 was then subjected to 10 successive rounds of subculture, and the urease activity of each generation remained stable (Fig. 1B).

### Single Factor Experiment

**Fig. 2:**
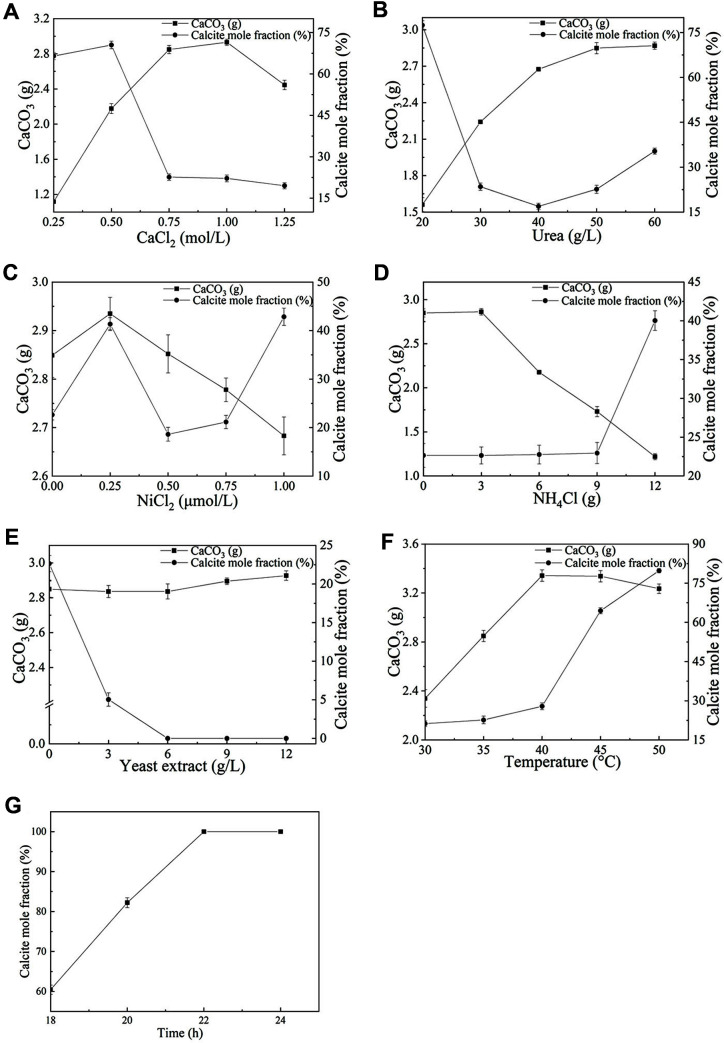
The effects of different factors including calcium chloride (**A**), urea (**B**), nickel chloride (**C**), ammonium chloride (**D**), yeast extract (**E**), temperature (**F**), and reaction time (G) on calcium carbonate precipitation and calcite ratio.

A total of six single factor experiments were conducted. The effect of different factors on the amount of precipitated calcium carbonate and the calcite ratio were shown in Fig. 2. Based on the amount of precipitated calcium carbonate as the index, the suitable range of factors was 0.5-1.0 mol/l calcium chloride, 40-50 g/l urea, 0-0.5 μmol/l nickel chloride, 0-3 g/l ammonium chloride, and 0-3 g/l yeast extract with a temperature of 35-45°C.

### Optimization of Biomineralization Conditions

The Plackett-Burman design was used to screen the factors that have significant effects on the biomineralization process, as shown in Table 1. The *p*-values for urea, calcium chloride, and temperature were all less than 0.05, indicating that these factors had significant effects on calcium carbonate precipitation. Furthermore, significant influence factors such as urea, calcium chloride and temperature had positive effects on the carbonate precipitation, while insignificant factors such as yeast extract, ammonium chloride and nickel chloride had negative effects.

Based on the results of the Plackett-Burman experimental design, a steepest climbing approach was applied as shown in Table 2. As can be seen in the table, the amount of precipitated calcium carbonate reached the maximum in test group 3. Therefore, the subsequent response surface optimization was performed by centering on the level of each factor.

Based on the results of the Plackett-Burman and steepest climbing designs, the Box-Behnken experimental design was applied. The experimental design and results are shown in Table 2. The regression equation was:

$$Y=3.31+0.45X_{1}+0.11X_{2}+0.23X_{3}+0.017X_{1}X_{2}+0.052X_{1}X_{3}+5.000\text{E-004}X_{2}X_{3}-0.44X_{1}^{2}-0.12X_{2}^{2}-0.24X_{3}^{2}$$

where, *X_1_*= CaCl_2_, *X_2_*= Urea, *X_3_*= Temperature.

The results of variance analysis and the significance test of the quadratic polynomial regression model are shown in Table 3. Results with *p*-values of less than 0.01 were considered highly significant. The *p*-value of the missing term was 0.1254 (*p* > 0.05), indicating that it was not significant. The R^2^ was 0.9978 and the adjusted R^2^ was 0.9939, indicating that the model had a good fit with the measured values. Moreover, the predicted R^2^ of 0.9677 indicated that the model had good predictability and could be used for the optimization of mineralization conditions. It was found that the primary items X_1_, X_2_, and X_3_, as well as the secondary items X_1_^2^, X_2_^2^, and X_3_^2^, were highly significant, while the interaction term X_1_X^3^ was significant. There was a clear interaction between the two factors.

**Fig. 3:**
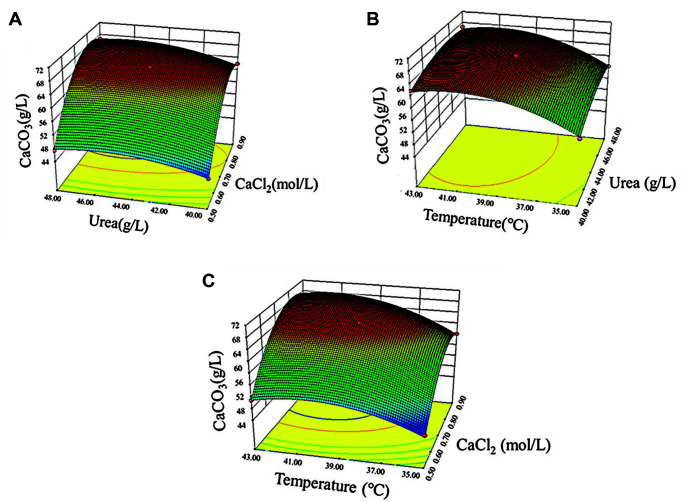
The response surface diagram depicting the influence of the interaction between two factors on the precipitation of calcium carbonate. (**A**) Urea and calcium chloride, (**B**) temperature and urea, (**C**) temperature and calcium chloride.

The three-dimensional response surface diagram of the interaction between the two factors and the precipitation of calcium carbonate was drawn according to the regression equation, as shown in Fig. 3. The model predicted that when the concentration of calcium chloride was 0.73 mol/l, the concentration of urea was 45 g/l, and the temperature was 40°C (marked as optimized condition 1), the amount of precipitated calcium carbonate was 68.08 g/l. The actual amount of calcium carbonate in the corresponding experiment was 67.32 g/l, indicating a high degree of agreement between the experimental and predicted values. Therefore, the calcium carbonate precipitation was increased by 18.15% under the optimized condition 1.

### Optimizing the Form of Calcium Carbonate Crystals

The three main crystal forms of calcium carbonate are aragonite, vaterite, and calcite, whereby the latter has the greatest stability, the highest density and the most compact structure [25]. Aragonite is an unstable phase, but it can be transformed into calcite by changing certain conditions. Therefore, based on the optimized condition 1, the conditions were further optimized to obtain a higher proportion of calcite. The effects of different factors on the calcite ratio were investigated as shown in Fig. 2. A lower calcium chloride concentration (0.25-0.5 mol/l), lower urea concentration (20 g/l), suitable nickel chloride concentration (0.25 μmol/l), higher ammonium chloride concentration (12 g/l) and high temperature (45-50°C) were beneficial to the formation of calcite, and the ratio of calcite crystals in calcium carbonate was greater. Therefore, the proportion of calcite might be increased by appropriately increasing the temperature and prolonging the mineralization time, without affecting the total yield of precipitated calcium carbonate. As shown in Fig. 2G, when the mineralization reaction time was extended to 22 h and the temperature increased to 45°C, a 100% calcite crystal form was obtained. Therefore, the optimized mineralization conditions (marked as optimized condition 2) included a calcium chloride concentration of 0.73 mol/l, urea concentration of 45 g/l, temperature of 45°C and reaction time of 22 h.

### Characterization of the Precipitates

**Fig. 4:**
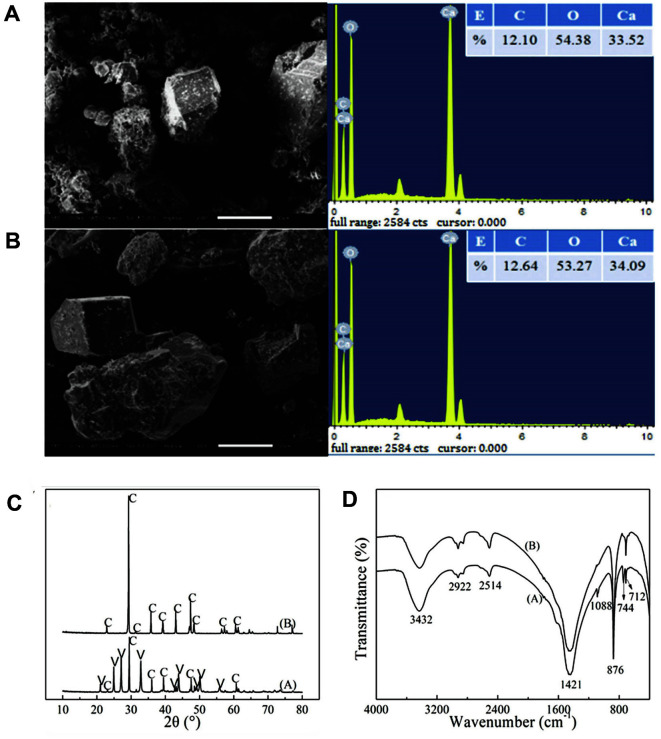
SEM and EDS analysis of calcium carbonate precipitates obtained under optimized condition 1 (**A**) and optimized condition 2 (**B**). XRD pattern (**C**) and FTIR pattern (**D**) of calcium carbonate precipitate. A and B stand for the precipitates obtained under optimized conditions 1 and 2, respectively. The letters C and V in Fig. 4C stand for calcite and vaterite.

SEM was used to observe the morphology of the precipitated calcium carbonate, and the elemental analysis of the sample micro-area was carried out using an EDS system. The surface morphology and main elemental composition of the precipitate crystals obtained under optimized conditions 1 and 2 are shown in Fig. 4. The crystals of the precipitate were spherical and ellipsoidal, while other components were rhomboid and irregular. The main elements were Ca, C, and O, and the ratio of atomic percentages converted from the ratio of weight percentages shown in Fig. 4 was close to 1:1:3, suggesting that both precipitate samples were calcium carbonate. Compared with condition 1, condition 2 increased the proportion of rhomboid and irregular block crystals.

The precipitated crystal forms were analyzed by XRD and FTIR. The XRD pattern is shown in Fig. 4C. According to the data of the PDF standard card (calcium PDF No: 00-005-0586, ball gangue PDF No: 00-033-0268), it was found that the precipitates obtained under optimized condition 1 showed characteristic peaks of both calcite and vaterite, whereby calcite accounted for 28.44%. By contrast, only the characteristic peak of calcite was detected under optimized condition 2. The FTIR results are shown in Fig. 4D. By comparison with the standard infrared spectrum of calcium carbonate, according to Andersen's report [26], the precipitates obtained under optimized condition 1 contained calcite and vaterite. The peaks at 2,514 cm^-1^, 1,421 cm^-1^, 876 cm^-1^ and 712 cm^-1^ were ascribed to calcite, while the peaks at 1,088 cm^-1^ and 744 cm^-1^ were ascribed to vaterite. In the precipitates obtained under optimized condition 2, there were only the calcite peaks at 2,514 cm^-1^, 1,421 cm^-1^, 876 cm^-1^ and 712 cm^-1^. The results of XRD and FTIR analysis were consistent, indicating that the precipitate obtained under condition 1 contained both calcite and vaterite crystals, while the precipitate obtained under condition 2 contained only calcite crystals.

### Repair Effect of Microbially Precipitated Calcium Carbonate on Concrete Specimens

The optimized mineralization conditions were used to repair small defects on the surface of the concrete specimens and large-sized cracks to detect the repair effect. Small defects were repaired by agar-loaded bacterial surface brushing. Cracks with a larger size were pre-filled with quartz sand and then the nutrient saline solution containing the bacteria was poured in to repair the cracks. The repair efficiency was evaluated by observing the surface healing effect as well as by comparing the water absorption and adhesion before and after repair.

**Fig. 5:**
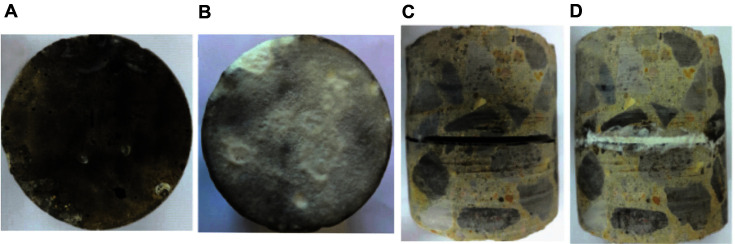
Visual observation of surface characteristerics of concrete specimens before and after repair. (**A**) and (**B**) were the surface states of concrete specimens with surface defects before and after being repaired. (**C**) and (**D**) were the states of concrete specimens with cracks before and after being repaired.

Figs. 5A and 5B show the surface state of specimens with defects before and after repair. The fact that the surface of specimens with defects after being repaired was covered with dense and white precipitates indicates that the concrete defects were effectively repaired. Figs. 5C and 5D show the states of specimens with cracks before and after repair. The cracks after repair were filled with quartz sand and calcium carbonate precipitation induced by the microorganisms resulting in a tight structure. Thus, the method developed in this study was able to repair larger cracks in concrete specimens.

**Fig. 6:**
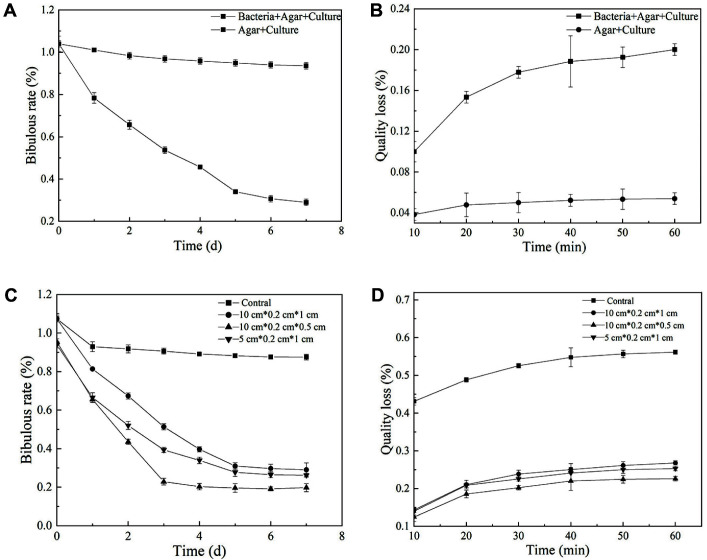
The changes of bibulous rate (**A**) and quality loss (**B**) of the specimens with surface defects after being repaired, and the specimens with concrete cracks after being repaired (C and D). In the control group, the specimen with surface defect and crack was both repaired without adding bacteria while other conditions remained the same with experimental group.

The water absorption of concrete specimens was tested after repair. Fig. 6A shows the changes in water absorption after the surface defects were repaired. The water absorption of the experimental group decreased by 71% after 7 days. In Fig. 6C, the water absorption of the experimental specimens with different crack sizes was also significantly reduced after repair, but the repair effect on cracks with smaller depth was better, resulting in an 80%reduction in bibulous rate.

The adhesion of concrete specimens was measured as shown in Fig. 6. When the specimens with defects were repaired and subjected to ultrasonic treatment, the mass loss of specimens gradually increased in the experimental group (Fig. 6B); however, the quality loss was within an acceptable range. The change of mass loss after crack repair is shown in Fig. 6D. With the prolongation of the ultrasonication time, the mass loss of specimens in the experimental group was significantly smaller than in the control group, and when other conditions were the same, specimens with smaller crack depth exhibited less mass loss. These results exhibited the same trend as the water absorption of the specimens.

## Discussion

In this study, *Sporsarcina pasteurii* ATCC 11859 was used as the starting strain for ARTP mutagenesis. Considering the MICP, the environmental pH of pores in concrete structures is usually alkaline and the application environment is harsh. However, ARTP mutagenesis could also work on proteins, nucleic acids and even whole cells of the strain to produce more mutant strains with superior traits. Following ARTP mutagenesis, strains with stronger alkali resistance and faster growth were selected in first-stage screening, and the mutant strains with better traits were selected for the next shake-flask fermentation screening. After screening and re-screening in shake flasks, six strains with higher urease activity were selected. Strain B11 exhibited urease activity of 38.66 ± 0.44 mM/min (Fig. 1A), which was 53.53% higher than the activity of the starting strain, The genetic stability was assessed by determining the yield of the enzyme in each generation of strain through subculture. Because the strain B11 could stably produce urease for more than 10 generations (Fig. 1B), and the variation coefficient of urease activity was only 1.12%, the mutant B11 could be considered genetically stable, indicating that ARTP is an efficient mutagenesis tool for spore-forming bacteria.

Microbially induced calcium carbonate deposition is a relatively complex process that is affected by many factors. To exclude biological factors, we used a fixed microbial concentration and mainly explored the effects of six abiotic factors on calcium carbonate deposition, including calcium chloride, urea, nickel chloride, ammonium sulfate, yeast extract and temperature (Fig. 2). Using those six factors as experimental variables, the microbially induced calcium carbonate deposition test was carried out with mutant strain B11, and the mineralization conditions were optimized using a Plackett-Burman design and response surface methodology to increase the amount of deposited calcium carbonate. According to the analysis, the factors that had significant effects on calcium carbonate deposition were calcium chloride, urea, and temperature.

Ca^2+^ accumulates mainly outside the cell in the metabolism of microorganisms [5]. Negatively charged molecules can lead to the adsorption of Ca^2+^ on the surface of bacterial cells, and the cell provides a nucleation site for the formation of calcium carbonate crystals. At sufficiently high CO_3_^2−^ concentrations, calcium carbonate deposition requires sufficient Ca^2+^. However, excessive Ca^2+^ can hinder the induction of calcium carbonate deposition [27]. Therefore, when it exceeded a certain range (1.0 mol/l), even if the Ca^2+^ concentration increased (Fig. 2A), it did not lead to a further increase of calcium carbonate precipitation [28, 29].

During microbially induced calcium carbonate deposition, microbial urease decomposes urea to produced CO_3_^2−^. Within a certain range, the increasing urea concentration leads to higher CO_3_^2−^ production by urease, which in turn leads to more precipitated calcium carbonate being formed (Fig. 2B). Some studies have shown that urea could induce the increase of urease expression [30-32]. Regulation of urease activity was a complex process, which would maintain urease activity at a relatively stable level. Therefore, the amount of calcium carbonate precipitates increased first and then remained unchanged as the urea concentration was further increased.

The main effects of temperature on microbially induced calcium carbonate deposition are exerted by influencing the activity of microbial urease and increasing the thermal motion of ions. An appropriate increase of the temperature can increase urease activity (Fig. 2F), enhancing the thermal motion of ions to accelerate ion exchange and calcium carbonate deposition [33].

Nickel chloride (Fig. 2C), ammonium sulfate (Fig. 2D), and yeast extract (Fig. 2E) were found to have less significant effects on calcium carbonate precipitation. A suitable concentration of nickel ions can increase urease activity, but higher concentration can inhibit urease activity [33]. Additionally, high concentration of NH_4_^+^ can also inhibite urease activity [34]. When NH_4_^+^ concentration increased and environmental pH decreased, it was not possible to provide a highly alkaline environment, which ultimately led to a decrease in calcium carbonate precipitation. Yeast extract provides nutrients for the microorganisms, but the results indicated that the addition of yeast extract had little effect on calcium carbonate precipitation.

In this study, the mutant B11 obtained by ARTP mutagenesis of *S. pasteurii* ATCC 11859 produced 56.98 g/l calcium carbonate under the initial conditions. Based on this, response surface methodology was used to improved the CaCO_3_ precipitation by 18.15% and increase calcium carbonate production 67.32 g/l.

Calcium carbonate has three main crystal forms, calcite, aragonite and vaterite. Among them, calcite had the greatest stability, the highest density and the most compact structure [35]. Therefore, based on the response surface optimization of the calcium carbonate precipitation, the conditions were further optimized to obtain a higher proportion of calcite. The influence of several factors on the ratio of calcite was explored.

When the concentration of calcium chloride was low, the ratio of calcite in calcium carbonate was high (Fig. 2A). It might be due to the fact that Ca^2+^ affected the nucleation and growth process of calcium carbonate. Among the two crystal forms of calcium carbonate, the solubility of calcite is lower than that of vaterite, and when the Ca^2+^ concentration was low (0.25-0.5 mol/l), calcite was preferentially formed, thus the content of calcite was high. When the Ca^2+^ concentration was increased, more vaterite was formed, so the content of calcite was reduced.

Additionally, a low urea concentration led to a high ratio of calcite (Fig. 2B). At high urea concentrations, the concentration of CO_3_^2-^ also increases, which may have favored the formation of vaterite. Additionally, a higher temperature increased the proportion of calcite. Because calcite is a thermodynamically stable phase, a high temperature can potentially transform the formed vaterite into the thermodynamically stable calcite, thereby increasing the calcite content.

As the concentration of ammonium chloride increased, the ratio of calcite also increased (Fig. 2D), probably because the high concentration of NH_4_^+^ inhibited the urease activity of the microorganism and reduced the amount of CO_3_^2-^ produced by the decomposition of urea, which in turn facilitated the formation of calcite. Nickel chloride promoted the activity of microbial urease and decomposition of urea to provide more CO_3_^2-^. However, a further increase of nickel chloride concentration would inhibit the urease activity, which was not conducive to the phase transformation of vaterite into calcite. When the concentration of nickel chloride was further increased, the urease activity was inhibited to a greater extent, which was conducive to the formation of calcite. Since calcite crystals are the most stable, dense and compact, it is beneficial to increase the proportion of calcite crystals by ensuring a large amount of calcium carbonate precipitation in practical applications. Considering the influence of the tested factors on the crystal form of calcium carbonate, increasing the temperature and extending the reaction time was adopted to increase the proportion of calcite without decreasing the total amount of precipitated calcium carbonate. Finally, the ratio of calcite was increased from 28.44 to 100% (Fig. 2G), which was confirmed by XRD, SEM, EDS, and FTIR characterization (Fig. 4).

After applying the optimized mineralization conditions to repair cracks in concrete specimens, it was found that small defects and cracks were covered with dense white calcium carbonate precipitation (Fig. 5), Moreover, the water absorption and mass loss were significantly lower than in the control group (Fig. 6). The added bacteria induced a large amount of calcium carbonate precipitation under sufficient nutrient conditions for growth, which reduced the pores of the specimen and improved its compactness [36]. This densification would reduce the penetration of water, and its adhesion was also improved with the increase of the specimen strength. For cracks of the same length and width, the repair effect was better on cracks with smaller depth. It is possible that deeper cracks would decrease the survival rate of microorganisms, and reduce the calcium carbonate precipitation, thereby weakening the repair effect.

## Conclusion

In order to apply microbial materials to repair cracks in concrete, strain mutagenesis and optimization of biomineralization condition were combined to increase the amount of calcium carbonate precipitation. Further improvement of the proportion of stable calcite in the precipitate was investigated to achieve better application in concrete repair. The maximum calcium carbonate production of 67.32 g/l in 100% calcite crystal form was achieved under the optimized conditions. After 7 days of treatment for specimens with defects and cracks under optimized mineralization conditions, the water absorption of the concrete specimens decreased by more than 70% compared with the control group, and the adhesion of the specimens was also improved significantly. This study provides a new protocol to achieve better performance of microbially induced calcium carbonate precipitation for the repair of concrete.

## Supplemental Materials

Supplementary data for this paper are available on-line only at http://jmb.or.kr.

## Figures and Tables

**Table 1:** Design and results of Plackett-Burman experiment.

Run	Yeast extract (g/l)	NH_4_Cl (g/l)	Urea (g/l)	CaCl_2_ (mol/l)	NiCl_2_ (μmol/l)	Temperature (°C)	CaCO_3_ （g/l）
1	3(1)	2(-1)	50(1)	0.5(-1)	0.25(-1)	36(1)	44.12
2	2(-1)	2(-1)	40(-1)	0.5(-1)	0.25(-1)	36(1)	40.78
3	2(-1)	2(-1)	50(1)	0.75(1)	0.375(1)	36(1)	52.06
4	3(1)	2(-1)	40(-1)	0.5(-1)	0.375(1)	45(-1)	45.84
5	3(1)	3(1)	50(1)	0.5(-1)	0.375(1)	45(-1)	49.14
6	2(-1)	2(-1)	40(-1)	0.75(1)	0.375(1)	45(-1)	53.96
7	2(-1)	3(1)	40(-1)	0.5(-1)	0.25(-1)	45(-1)	45.94
8	3(1)	3(1)	40(-1)	0.75(1)	0.375(1)	36(1)	44.06
9	3(1)	3(1)	40(-1)	0.75(1)	0.25(-1)	36(1)	44.26
10	3(1)	2(-1)	50(1)	0.75(1)	0.25(-1)	45(-1)	66.18
11	2(-1)	3(1)	50(1)	0.5(-1)	0.375(1)	36(1)	43.56
12	2(-1)	3(1)	50(1)	0.75(1)	0.25(-1)	45(-1)	65.9
T-test	-1.12	-1.34	6.07	7.51	-2.44	7.68	
Pr>|T|	0.313	0.237	0.002	0.001	0.059	0.001	
Significant ordering	6	5	3	2	4	1	

**Table 2:** Design and results of the steepest ascent and Box-Behnken experiments.

	Run	CaCl_2_ (mol/L)	Urea (g/l)	Temperature (°C)	CaCO_3_ (g/l)
Steepest ascent experiment	1	0.5	40	35	44.46
	2	0.6	42	37	51.72
	3	0.7	44	39	66.26
	4	0.8	46	41	65.98
	5	0.9	48	43	66.46
	6	1.0	50	45	66.34
Box-Behnken experiment	1	0.5(-1)	40(-1)	39(0)	44.18
	2	0.5(-1)	48(1)	39(0)	47.4
	3	0.9(1)	40(-1)	39(0)	62.16
	4	0.9(1)	48(1)	39(0)	66.7
	5	0.7(0)	40(-1)	35(-1)	51.96
	6	0.7(0)	40(-1)	43(1)	61.78
	7	0.7(0)	48(1)	35(-1)	56.62
	8	0.7(0)	48(1)	43(1)	66.48
	9	0.5(-1)	44(0)	35(-1)	40.92
	10	0.9(1)	44(0)	35(-1)	56.28
	11	0.5(-1)	44(0)	43(1)	47.02
	12	0.9(1)	44(0)	43(1)	66.54
	13	0.7(0)	44(0)	39(0)	66.62
	14	0.7(0)	44(0)	39(0)	66.18
	15	0.7(0)	44(0)	39(0)	65.96

**Table 3:** Analysis of variance for regression model.

Source of Variance	Sum of squares	Degree of freedom	Mean square	F	*Pr*>F	Significance
Model	3.03	9	0.34	254.35	<0.0001	**
X_1_	1.63	1	1.63	1231.02	<0.0001	**
X_2_	0.092	1	0.092	69.29	0.0004	**
X_3_	0.41	1	0.41	307.07	<0.0001	**
X_1_X_2_	1.09E-03	1	1.09E-03	0.82	0.4057	
X_1_X_3_	0.011	1	0.011	8.18	0.0354	*
X_2_X_3_	1.00E-06	1	1.00E-06	7.57E-04	0.9791	
X_1_^2^	0.72	1	0.72	544.69	<0.0001	**
X_2_^2^	0.049	1	0.049	37.32	0.0017	**
X_3_^2^	0.21	1	0.21	156.35	<0.0001	**
Residual	6.61E-03	5	1.32E-03			
Lack of Fit	6.04E-03	3	2.02E-03	7.14	0.1254	
Pure error	5.65E-03	2	2.82E-04			
Total	3.03	14				

Note: R^2^ = 0.9978, Adj R^2^ = 0.9939, Pred R^2^ = 0.9677, **p < 0.01 is extremely significant; *p < 0.05 significant. *X_1_* = CaCl_2_, *X_2_* = Urea, *X_3_* = Temperature.
