# Supplementary material for: Improvement of Biomineralization of Sporosarcina pasteurii as Biocementing Material for Concrete Repair by Atmospheric and Room Temperature Plasma Mutagenesis and Response Surface Methodology
Source: J Microbiol Biotechnol. 2021 Aug 3;31(9):1311–22. doi: 10.4014/jmb.2104.04019 (PMC9705896; doi:10.4014/jmb.2104.04019)
Supplement: Supplementary file 1 [file jmb-31-9-1311-supple.pdf]

https://doi.org/10.4014/jmb.2104.04019

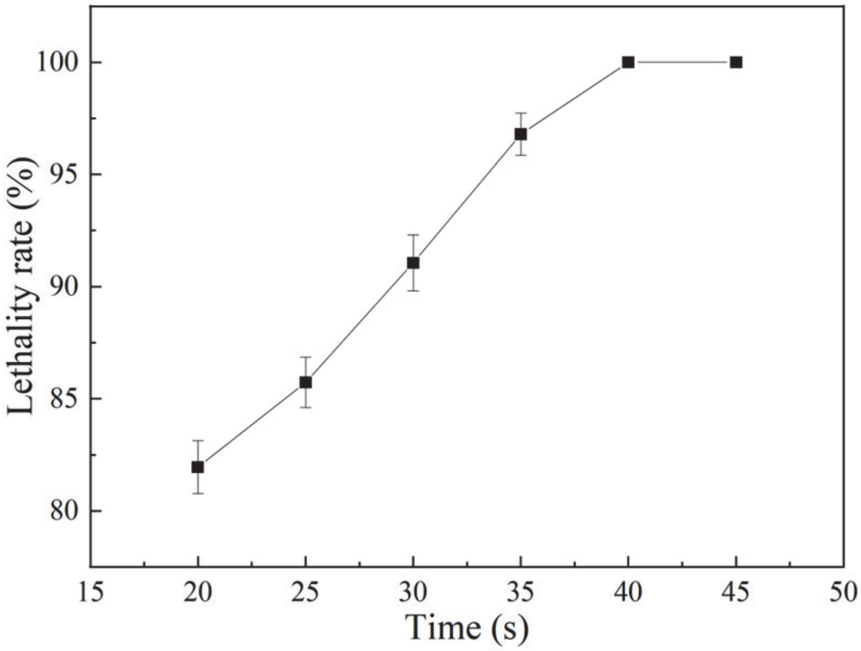

Fig. S1 Lethality rate of ARTP for *Sporosarcina pasteurii* ATCC 11859.

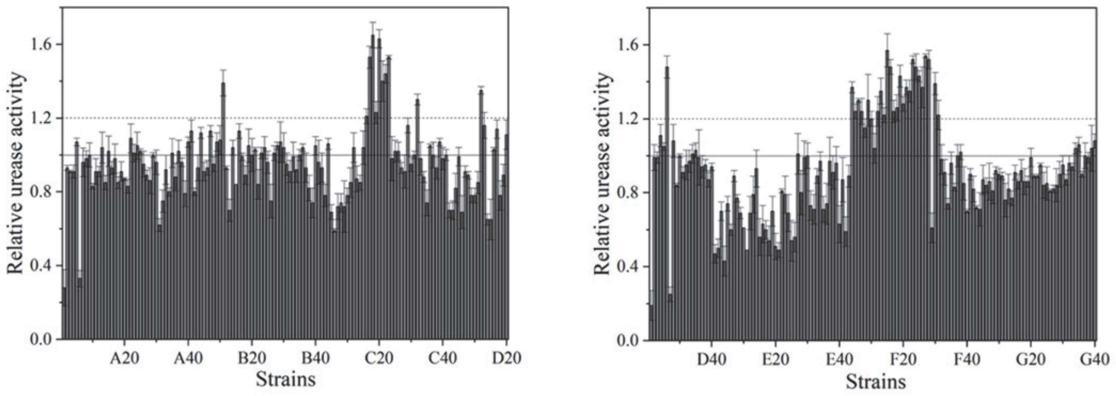

Fig. S2 The result of preliminary screening with flask-shaking. The solid line in the picture shows the urease activity of *Sporosarcina pasteurii* ATCC 11859, while the dotted line indicates that the urease activity is increased by 20% compared with *Sporosarcina pasteurii* ATCC 11859.

Table S1. The results of secondary screening with flask fermentation

Strain	Urease activity (mM.min^{-1}）	OD_{600}	Unit urease activity（mM.min^{-1}.$OD_{600}$$^{-1}$）
B9	37.03±0.26	6.02±0.04	6.15±0.08
B11	38.66±0.44	5.66±0.06	6.83±0.08
C12	35.69±0.24	5.86±0.04	6.09±0.06
C20	38.07±0.26	5.71±0.02	6.67±0.07
C23	35.55±0.44	5.55±0.05	6.41±0.14
D26	36.59±0.26	5.97±0.04	6.13±0.05
ATCC 11859	25.18±0.25	4.92±0.05	5.12±0.10
